# Development of Wax-Incorporated Emulsion Gel Beads for the Encapsulation and Intragastric Floating Delivery of the Active Antioxidant from *Tamarindus indica* L.

**DOI:** 10.3390/molecules21030380

**Published:** 2016-03-22

**Authors:** Sitthiphong Soradech, Intira Petchtubtim, Jeerayu Thongdon-A, Thanchanok Muangman

**Affiliations:** Department of Pharmaceuticals and Natural Products, Thailand Institute of Scientific and Technological Research, Pathum Thani 12120, Thailand; Sitthiphong@tistr.or.th (S.S.); Intira@tistr.or.th (I.P.); Jeerayu@tistr.or.th (J.T.-A)

**Keywords:** tamarind seed extract, antioxidant activity, mitochondria toxicity, ionotropic gelation technique, emulsion gel beads, encapsulation

## Abstract

In this study, tamarind (*Tamarindus indica* L.) seed extracts with potential antioxidant activity and toxicity to cancer cells were developed as functional foods and nutraceutical ingredients in the form of emulsion gel beads. Three extracts were obtained from ethanol and water: TSCH50, TSCH95 and TSCH. All extracts exhibited high potential for superoxide anion scavenging activity over the IC_50_ range < 5–11 µg/mL and had no toxic effects on normal cells, however, the water extract (TSCH) was the most effective due to its free radical scavenging activity and toxicity in mitochondrial membranes of cancer cells. Next a study was designed to develop a new formulation for encapsulation and intragastric floating delivery of tamarind seed extract (TSCH) using wax-incorporated emulsion gel beads, which were prepared using a modified ionotropic gelation technique. Tamarind seed extract at 1% (*w*/*w*) was used as the active ingredient in all formulations. The effect of the types and amounts of wax on the encapsulation efficiency and percentage of the active release of alginate gel beads was also investigated. The results demonstrated that the incorporation of both waxes into the gel beads had an effect on the percentage of encapsulation efficiency (%) and the percentage of the active ingredient release. Furthermore, the addition of water insoluble waxes (carnauba and bee wax) significantly retarded the release of the active ingredient. The addition of both waxes had a slight effect on drug release behavior. Nevertheless, the increase in incorporated waxes in all formulations could sustain the percentage of active ingredient release. In conclusion, wax-incorporated emulsion gel beads using a modified ionotropic gelation technique could be applied for the intragastric floating delivery and controlled release of functional food and nutraceutical products for their antioxidant and anticancer capacity.

## 1. Introduction

Gel beads or spheres represent a new drug delivery system formulation that can be prepared from natural biopolymers, such as sodium alginate, chitosan or pectin. Sodium alginate is a non-toxic, biodegradable and naturally occurring polysaccharide derived from marine brown algae, which contain bacterial species [1,2]. Sodium alginate is a water soluble sodium salt of alginic acid containing a linear polymer composed of 1,4-linked β-d-mannuronic acid (M) and α-d-guluronic acid (G) residues that forms a reticulated structure [3,4]. Alginate gel beads are the result of cross-links with divalent or polyvalent cations to form an insoluble mesh [5]. Calcium and zinc cations have been reported for the cross-linking of acid groups of alginate and have been used for the controlled release of active ingredients [5,6,7]. The benefits are a low cost and an abundance of sources, as well as excellent biocompatibility and total degradation without hazardous by-products. The encapsulation efficiency, morphology, swelling, floating properties and drug release of gel beads were also studied [7]. It was noted, however, that alginate gel beads could not prolong the gastric retention of active ingredients [1,8,9,10]. New formulations were attempted based on different processes, such as floating, expansion/plug type, high density or adhesion to mucosa. Research on floating systems, in particular, has been widely conducted [9]. Nevertheless, the floating system had no effect on the motility of the GI tract. Immediate floating could be obtained when the density was low at the beginning, for example, when provided by the entrapment of air or by the incorporation of low density materials, such as oils and foam powders [7,11]. Therefore, the development of new formulations using a novel drug delivery system for encapsulation of the potential ingredient is important to develop new technology for nutraceutical and functional food applications.

*Tamarindus indica* L. (commonly called tamarind, family Fabaceae, subfamily Caesalpiniaceae) is a tropical evergreen tree native to fertile areas throughout Africa and Southern Asia. It is widely cultivated as an ornamental tree and for its acidic fruits used in food materials and is a popular component of many decoctions used as health remedies [12,13,14,15]. Recently, tamarind research has concentrated on its seeds, which are a waste product from consumption. There have been several attempts to utilize tamarind seeds, especially as food, pharmaceutical and cosmetic ingredients [15]. The tamarind seed coat also contains active antioxidants, e.g., phenolics, tannins and flavonoids [16], and its extracts possess lipid peroxidation reduction, anti-tyrosinase collagen stimulating, anti-microbial, anti-inflammatory, anti-diabetic and anti-hyperlipidemic activities [12,14,17,18,19,20,21,22,23]. However, there is no information about the effect of tamarind seed extract on cancer cells, including possible cytotoxic and apoptotic activities.

Hence, in this study, tamarind seeds were extracted with various solvents, and the antioxidant activity and apoptosis activity of these extracts was determined via mitochondrial toxicity. The effective conditions for tamarind seed extraction were selected, and a study was designed to develop a new formulation for encapsulation and intragastric floating delivery using wax-incorporated emulsion gel beads.

## 2. Results and Discussion

### 2.1. Tamarind Seed Extracts

The yields of the three extracts—50% (TSC50) and 95% (TSC95) ethanol and water (TSCH) extract—were 19.809%, 49.242% and 53.879%, respectively. The highest extraction yield was thus obtained for the tamarind seed coat extract with water (TSCH). This result is in the line with Nakchat *et al.* [17], who showed that TSCE-W (water extract) had the highest phenolic content, much higher than TSCE-E (70% ethanol). This result indicates that water was suitable for tamarind seed coat extraction.

### 2.2. Total Phenolic Content of Extracts

Total phenolic content is one of the key characteristics of *Tamarindus indica* L. extract and was observed using thin layer chromatography (data not shown). The result was in agreement with another study [16], which found that the phytochemical constituents of *Tamarindus indica* L. extracts include phenolics, flavonoids, alkaloids, tannins, cyanogenic glycosides and anthroquinones. Total phenolic content was determined by UV-Visible spectrophotometry at 760 nm and is expressed as milligrams of gallic acid equivalents per g of extract, as shown in Table 1. The results indicated that the total phenolic contents of the three extracts, including 50% (TSC50) and 95% (TSC95) ethanol and water (TSCH) extract, were 23.69 GAE mg/g extract, 43.98 GAE mg/g extract and 39.39 GAE mg/g extract, respectively.

### 2.3. Superoxide Anion (O_2_^•−^) Scavenging Activity of Extracts

The scavenging activity of the extracts exhibited a dose-response relationship, as shown in Table 1. All the ethanoic extracts showed strong O_2_^•−^ scavenging activity in the IC_50_ < 5–11 µg/mL value range. The inhibition was greater than 50%, even at a dose less than 5 µg/mL of both the TSC95 and TSCH extracts. This observation is fairly common in plants because few non-polar compounds are able to act as potent antioxidants [12,22,24]. However, Tsuda *et al*. reported that the methanol and the ethyl acetate extracts of tamarind had marked activity, indicating that tamarind has both polar and specific non-polar anti-oxidative substances [14].

### 2.4. Mitochondrial Dehydrogenase Activity Assay (WST Assay) of Extracts

The effect of tamarind seed extracts on mitochondrial toxicity was measured in five cell line cultures. Mitochondrial succinate dehydrogenase activity was represented for alteration of mitochondrial function. As shown in Table 2, cells were treated with tamarind seed coat extracts in the range of 15 to 1000 µg/mL for 24 h, and the IC_50_ values represented the concentrations of extracts that inhibited mitochondrial succinate dehydrogenase activity by 50%. All extracts occurred in the absence of any effect on enzyme activity, except for TSCH extract. Caco2 cells showed the highest effect on all of the tamarind seed coat extracts. The TSCH extract had a strong effect on cancer cell lines; IC_50_ values were found to be 75.27, 15.06 and 100.37 µg/mL for HepG2, HeLa and Caco2, respectively. The IC_50_ values of TSCH extract had little effect on normal cells, such as HK-2. Additionally, Nakchat *et al*. [23] have reported that tamarind seed coat extracted with water did not affect normal human foreskin fibroblast cells (CCD-10664sk). Moreover, polysaccharide PST001 isolated from tamarind seed kernel had an anti-proliferative effect on some cancer cells (A549, KB and DLA) based on the inhibition of the mitochondrial dehydrogenase activity [25].

### 2.5. Mitochondrial Membrane Potential (MMP Assay) of Extracts

**Figure 1:**
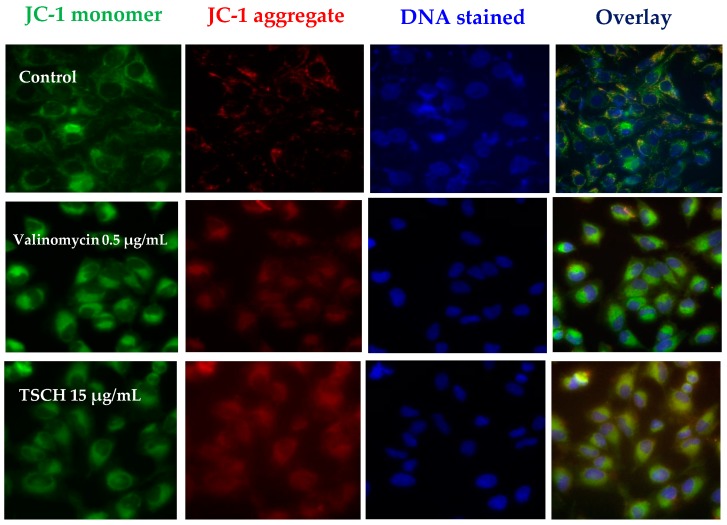
Fluorescence image of HeLa cells labeled with JC-1 and DNA stain Hoechst 3342 (blue). Cells were treated with valinomycin (0.5 µg/mL) and water extract of tamarind seed coat (TSCH) at concentrations of 5 and 15 µg/mL. Left and middle left panels show aggregate (red) and monomer (green) fluorescence, respectively. The right panels show the overlay of the two images; in this case, the orange/yellow color denotes co-localization of the red and green fluorescence signals.

**Figure 2:**
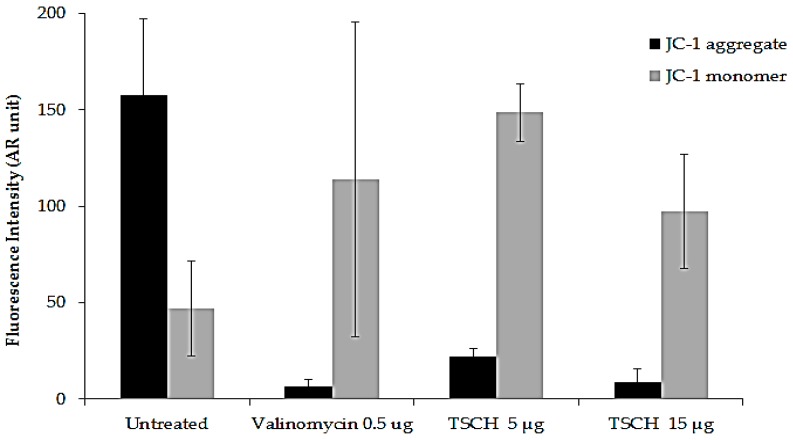
Effect of the water extract of tamarind seed coat (TSCH) on JC-1 fluorescence intensity. HeLa cells were incubated with TSCH at 5 and 15 µg/mL and compared with 0.5 µg/mL valinomycin (chemotherapeutic agent). Fluorescence intensities were analyzed after 30 min of JC-1 incubation. Values represent the mean ± S.D. of 10,000 cells.

The effect on mitochondrial membrane potential is important in the detection of apoptosis. Mitochondrial membrane potential was detected with JC-1 staining. In healthy cells, JC-1 accumulates in the mitochondria as JC-1 aggregates with intense red fluorescence, whereas unhealthy cells or cells undergoing apoptosis show only green fluorescence. Untreated HeLa cells exhibited strong aggregation of red fluorescence and slightly significant green (Figure 1). The degradation of mitochondrial membrane potential (green) was profound in HeLa cells treated with valinomycin. In this study, HeLa cells were treated with TSCH at concentrations of 5 and 15 µg/mL. The results demonstrated that the green fluorescence was more intense than the red fluorescence. The intensities of JC-1 aggregates and monomers are shown with arbitrary units in Figure 2. The average ratio of mitochondrial JC-1 aggregate intensity (red) and JC-1 monomer intensity (green) decreased in the following order: untreated cells (3.35) > TSCH 5 µg/mL (0.15) > TSCH 15 µg/mL (0.09) > valinomycin (0.06). Untreated cells (healthy cells) had the highest ratio of red/green. The ratio of red/green intensity in cells treated with valinomycin was similar to that obtained with 15 µg/mL TSCH extracts.

### 2.6. Conventional Gel Beads and Wax-Incorporated Emulsion Gel Beads

**Figure 3:**
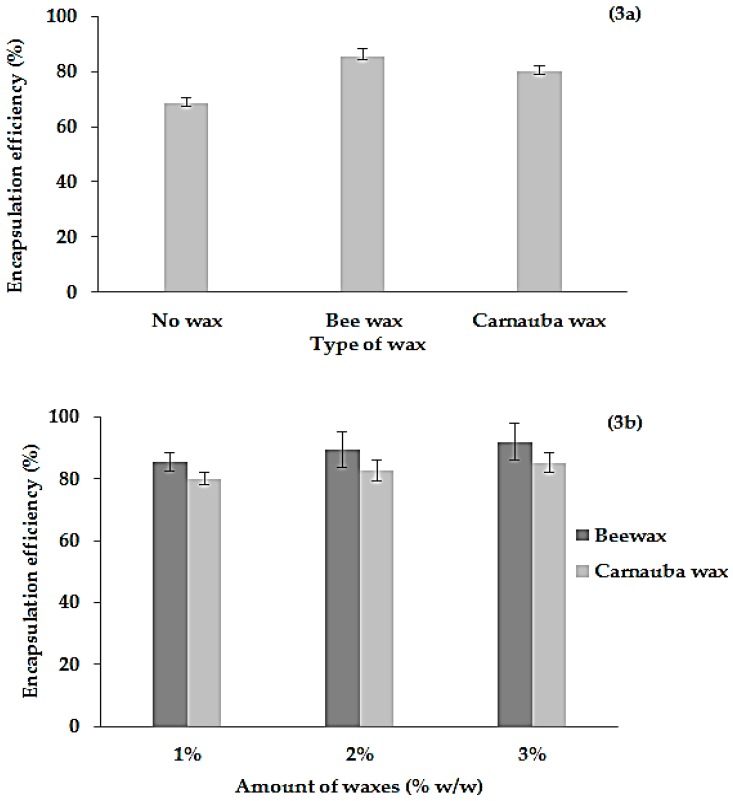
Effect of different types (**3a**) and amounts (**3b**) of waxes on the percentage of encapsulation efficacy of emulsion gel beads containing tamarind seed extract.

**Figure 4:**
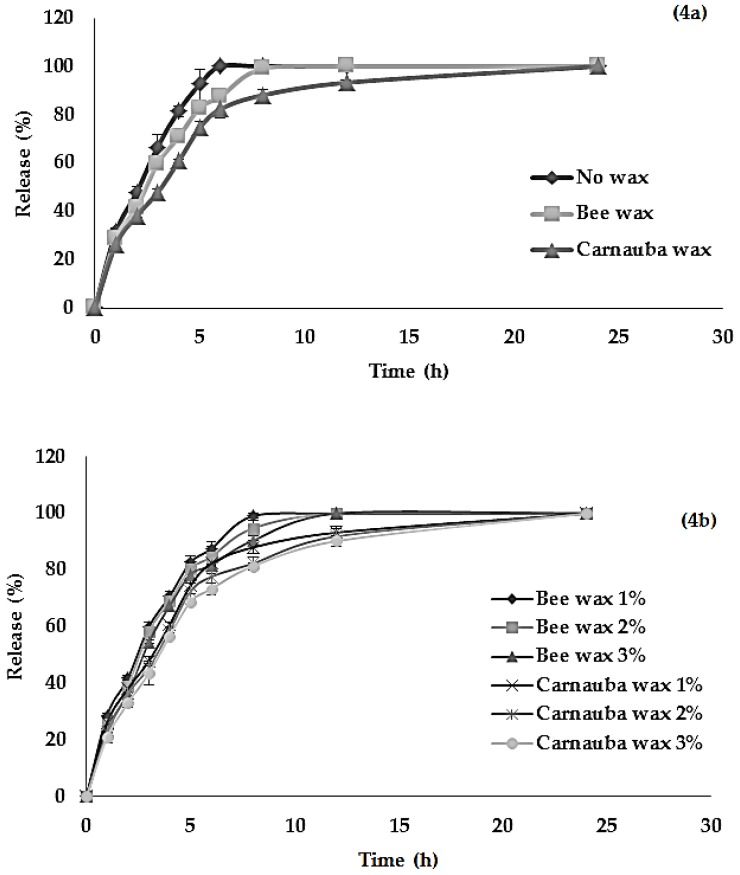
Effect of types (**4a**) and amounts (**4b**) of waxes on the percentage of active release from alginate gel beads containing tamarind seed extract.

The water extract (TSCH) was the most effective one due to its highest free radical scavenging activity and toxicity in the mitochondrial membrane of cancer cells. Therefore, the tamarind seed water extract was selected for further study of the development of new formulations for the encapsulation and intragastric floating delivery of tamarind seed extract by using wax-incorporated emulsion gel beads, which were prepared by a modified ionotropic gelation technique. Sodium alginate could emulsify the mixture of aqueous tamarind seed extract solution, as a result of the surface active ability of sodium alginate that could reduce the interfacial tension between the oil phase and water phase or steric and mechanical stabilization mechanisms. The tamarind seed extract at 1% (*w*/*w*) was used as the active ingredient in all formulations. The emulsion-gel beads were gelled by the action of calcium chloride. The molten wax was dispersed in the homogenized emulsion mixture of alginate. The wax and tamarind seed extract were heated at the same temperature and then mixed until a homogenous mixture was obtained. Finally, the hot-melted mixture was extruded into calcium chloride solution. Spherical beads of wax-incorporated emulsion gel were obtained by hot melt extrusion and ionotropic gelation. The effect of the types and amounts of waxes on the mean diameter, total phenolic content, percentage of encapsulation efficiency and percentage of active release of emulsion gel beads was also investigated, as shown in Table 3 and Figure 3 and Figure 4.

### 2.7. Particle Size of Gel Beads

The mean diameters of the active-loaded gel beads and wax-incorporated emulsion gel beads are shown in Table 3. The mean diameter of the tamarind seed extracts loaded gel beads ranged between 2.2 and 2.5 mm. The mean size of the beads changed insignificantly when the amount of wax was changed, which agreed with a previous report, in which the type of additives used (e.g., waxes or polymers) had an insignificant influence on the mean diameter of the emulsion gel beads [1].

### 2.8. Percentage of Encapsulation Efficiency (% EE)

In this study, total phenolic content was chosen as the model of the active ingredient for determination of the percentage of encapsulation efficiency (% EE) of tamarind seed extract. The total phenolic content of the extracts from the beads was in the range of 27.01 mg GAE mg/g extract to 36.26 mg GAE mg/g extract (Table 3). Moreover, the effect of different types and amounts of waxes on the percentage of encapsulation efficacy (% EE) of emulsion gel beads was also studied by the determination of the phenolic content, as shown in Figure 3a,b. The result demonstrated that the % EE of the active ingredients from tamarind seed extract increased with the incorporation of waxes into the formulation. The % EE in all formulations was 68.56%, 85.43% and 80.12% without wax, with bee wax and with carnauba wax, respectively (Figure 3a). The addition of both waxes into the gel beads showed a significant (*p* < 0.05) change in % EE of the emulsion gel beads compared with the gel beads without wax. The maximum of 85.43% active ingredients of % EE was obtained in emulsion gel beads containing bee wax because both waxes were natural complex lipid materials consisting of different amounts of primarily acid esters, *i.e.*, free acids, fatty alcohols, and hydrocarbons [7,26]. The change in % EE was associated with a change in the total phenolic content of the different beads. Sriamornsak [7,26] reported that white bee wax contains a higher percentage of free fatty acids, resulting in a lower percentage of fatty esters than carnauba wax.

The effects of the amounts of both waxes on the % EE of emulsion gel beads are shown in Figure 3b. The results indicated that the increase in the amounts of both waxes contributed to a significant (*p* < 0.05) increase in % EE compared with conventional gel beads. The increase in the amount of wax contributed to the decrease in the diameter of the emulsion gel beads, resulting in an increase in the % EE of the gel beads. This result was similar to that of another study [2], which found that the entrapment efficiency of metformin was increased with an increase in the wax concentration and that the percentage entrapment efficiency was 83.28% to 94.35%. However, the % EE of the present study was not significantly changed (*p* < 0.05) when the amount of wax was increased from 1% to 3%. The % EE of gel beads increased from 85.43% to 92.06% when the amount of white wax increased from 1% to 3%, whereas the % EE of gel beads increased from 80.12% to 85.32% when the amount of carnauba wax increased from 1% to 3%, respectively. Between both waxes, bee wax of all concentrations had a % EE higher than carnauba wax due to differences in the structures of the waxes.

### 2.9. Percentage of Active Ingredient Release

The floating delivery system is a gastroretentive delivery system, and the use of floating delivery enhances the efficiency and controls the delivery of many active ingredients that have pH-dependent solubility and instability at intestinal pH [27,28]. In this study, the extracts had potential for high antioxidant and anticancer capacity. Therefore, the release behavior of emulsion gel beads was studied to improve the delivery and performance of the antioxidant activity in the gastrointestinal tract over an extended period of time. The active ingredient release studies were performed only when the beads floated in simulated gastric fluid (SGF, pH 1.2) to determine the suitability of the beads as an intragastric floating delivery system. Simulated gastric fluid can be used to test drug absorption in the gastrointestinal tract, e.g., the stomach, which is a highly variable procedure, and prolonging gastric retention of the dosage form extends the time for drug absorption [27,28,29]. The active ingredient release profiles were presented by plotting the amount released against time.

The effects of various types and amounts of waxes on the active ingredient release profiles of emulsion gel beads is shown in Figure 4a,b. At the end of the first hour, active release was 31.24%, 28.74% and 26.21% for gel beads with bee wax, carnauba wax and without wax, respectively (Figure 4a). The slow release profile of the active ingredient was also reported for floating beads that contained white wax and carnauba wax in SGF compared with conventional gel beads. The results were due to the hydrophobic nature of waxes for dispersion in the structure of emulsion gel beads. The matrix with more hydrophobicity consequently delayed the diffusion of drug from the beads [1,7]. However, the different types of hydrophobic wax had a slight effect on active release behavior as a result of the similar structures of bee wax and carnauba wax [1,7]. Sriamornsak *et al*. [7] reported that both waxes were natural complex lipid materials consisting of different amounts of primarily acid esters, free acids, fatty alcohols, and hydrocarbons. Compared with beeswax, carnauba wax significantly retarded (*p* < 0.05) active ingredient release more effectively at the end of the first hour due to the higher percentage of free fatty acid and the lower percentage of fatty esters of white wax compared with without wax, and the result was in agreement with other studies [11].

The effect of the amount of waxes on the active ingredient release profiles of emulsion gel beads is presented in Figure 4b. As the wax concentrations increased, the active release from the floating beads decreased due to the structure and characteristics of the wax. The result agreed with Cao *et al*. [30], who also demonstrated that the release rate from lipophilic matrix tablets decreased when the amount of lipophilic excipients (stearyl alcohol, stearic acid and/or carnauba wax) increased. The increased amounts of carnauba wax were released more slowly than the increased amount of beeswax as a result of the increase in the water resistance of wax. A similar influence was exerted by an increase in the amount of wax on active ingredient release from gels based on different waxes. This study developed floated beads using the formulation of wax incorporated emulsion gel beads to enhance the efficiency and control the delivery of many active ingredients that had pH-dependent solubility and instability at intestinal pH. This method could be applied to functional food and nutraceutical products due to their antioxidant and anticancer capacity.

## 3. Materials and Methods

### 3.1. Materials

Sodium alginate and calcium chloride, (Sigma Aldrich, St. Louis, MO, USA), olive oil, carnauba wax and white wax (Nam Siang Co., Ltd., Bangkok, Thailand) were used as received. Tamarind seed extract was harvested from Agricultural and Food Technology Department, Thailand Institute of Scientific and Technological Research. All other chemicals were of standard pharmaceutical grade.

### 3.2. Extraction Conditions

Tamarind seeds from sweet Thai tamarind were purchased from a local market in Bangkok. The seed coats were mechanically separated from the germ. Seeds were ground using a mechanical grinder several times until a homogenous powder was obtained. Solvent extraction was performed by stirring with 50% and 95% ethanol for 48 h and filtering through Whatman No. 1 filter paper. The solvent of the extracts was evaporated under reduced pressure (34–36 kPa) using a rotary vacuum-evaporator at 40 °C, and the contents were freeze-dried. Water extraction was performed similarly to solvent extraction. The freeze-dried extracts were used directly for the assessment of SOD-like activity and mitochondrial toxicity assays.

### 3.3. Determination of Total Phenolic Content

Total phenolic contents were determined by the Folin-Ciocalteau assay using gallic acid [12] as the standard solution. Sample or standard solution (0.1 mL) was added to a 25 mL test tube containing 8.4 mL of distilled water. Folin-Ciocalteau reagent (0.5 mL) was added to the mixture and shaken for 5 min before adding 1 mL of 20% Na_2_CO_3_ solution. The mixture was shaken before incubating in the dark for 1 h at room temperature. Absorbance was measured against the reagent blank at 760 nm.

### 3.4. Determination of Superoxide Anion (O_2_^•−^) Scavenging Activity

Scavenging activity is based on the reduction rate of O_2_^•−^ by superoxide dismutase (SOD). O_2_^•−^ was generated in the system by xanthine oxidase (XO) activity. Water-soluble tetrazolium salt produced a water-soluble formazan dye upon reduction with a superoxide anion. The rate of the reduction with O_2_^•−^ is linearly related to XO activity and is inhibited by SOD. Therefore, the inhibition activity of SOD or SOD-like materials can be determined by a colorimetric method. Seed coat extract was added (20 µL) to each well sample and blank in 96-well plates and was mixed with 200 µL of the working solution, followed by adding 20 µL of enzyme working solution to each sample and blank. The wells were mixed thoroughly and incubated at 37 °C for 20 min. After incubation, absorbance was determined at 440 nm. SOD activity was expressed as the rate of inhibition (%).

### 3.5. Determination of Mitochondrial Toxicity

#### 3.5.1. Cell Culture and Reagents

Different cell lines were used in the experiment, including cancer cells and normal cells. Human colon adenocarcinoma (Caco-2) cells, human liver hepatocellular carcinoma (HepG2) cells, cervix adenocarcinoma (HeLa) cells, human kidney proximal tubular (HK-2) cells, and mouse subcutaneous connective tissue (L929) cells were used to determine mitochondrial toxicity. All cells were grown in DMEM medium and were supplemented with 10% (*v*/*v*) fetal bovine serum, except for L929, which was grown in MEM medium supplemented with 10% (*v*/*v*) horse serum in a humidified incubator at 37 °C and 5% CO_2_.

#### 3.5.2. Mitochondrial Dehydrogenase Activity Assay (WST Assay)

This assay depends on the cleavage of the soluble tetrazolium salt (WST-1, Roche, Mannleim, Germany) to form water soluble formazan via the action of mitochondrial succinate dehydrogenase found in visible cells. Cells were seeded in 96-well plates and were cultured for 24 h in a 5% CO_2_ incubator at 37 °C prior to treatment with various concentrations of Tamarind seed coat extracts. After 24 h of treatment, the medium in each well was replaced with 100 µL of new medium, and 10 µL of WST solution was added, following the assay kit instruction. Cells were incubated in a 5% CO_2_ incubator at 37 °C for 30 min. Cell viability was quantified by a spectrophotometer at 450 nm.

#### 3.5.3. Mitochondrial Membrane Potential Assay (MMP Assay)

The MMP assay was performed by the fluorescence method. Mitochondrial membrane potential can be used to detect apoptosis using 5,5′,6,6′-tetrachloro-1,1′,3,3′-tetraethylbenzimida-zolylcarbocyanine iodide (JC-1), a cationic fluorescence dye. JC-1 dye accumulates throughout the cell cytosol as a monomer, emitting primarily green fluorescence. In healthy, energized cells, the mitochondria′s negative charge established with an intact membrane potential allows the lipophilic dye to enter the mitochondrial matrix and accumulate. The increasing mitochondrial matrix concentration of the dye forms J-aggregates that exhibit red/orange fluorescence. Due to mitochondrial damage or loss of membrane potential, the dye cannot accumulate in the mitochondria, which is indicated by a lack of orange fluorescence. Hela cells at 1 × 10^5^ were grown and incubated with 5 and 15 µg/mL of tamarind seed coat water extract for 24 h. Valinomycin was used as a positive control and was incubated at 0.5 µg/mL with HeLa cells for 4 h. The extract was removed, and trypsinized cells were incubated with JC-1 dye solution (JC-1, Sigma Aldrich) at 37 °C and 5% CO_2_ for 30 min. After removing the dye, the cells were washed by PBS and were resuspended before being analyzed using a high content imaging fluorescence microscope (INCell Analyzer 2200, GE HealthCare, Seattle, WA, USA). The generated fluorescent products were visualized using double-field fluorescence with fluorescein isothiocyanate (FITC, 475/512 nm) and Texas Red (575/620 nm) filters. Fluorescent intensity was analyzed using the software IN Cell Developer Toolbox V 1.9.1 (GE HealthCare, Seattle, WA, USA). The ratio of red fluorescent intensity and green fluorescent intensity was calculated and presented as the mean + S.D.

### 3.6. Preparation of Conventional Calcium Alginate Gel Beads and Wax-Incorporated Alginate-Based Emulsion Gel Beads

Among all extracts, the water extract (TSCH) was selected for further study of the development of a new formulation for the encapsulation and intragastric floating delivery of tamarind seed extract using wax-incorporated emulsion gel beads, which were prepared from a modified ionotropic gelation technique. The preparation of conventional calcium alginate gel beads and wax-incorporated emulsion gel beads is discussed in Section 3.6.1 and Section 3.6.2.

#### 3.6.1. Preparation of Conventional Calcium Alginate Gel Beads

Calcium alginate gel beads were prepared by the ionotropic gelation method, which was adapted from Sriamornsak *et al*. [1,7]. First, 2% *w*/*w* alginate was dispersed in water with agitation, and the concentration of tamarind seed extract at 1% *w*/*w* was dispersed in alginate solution to make a 100 g solution. Then, the dispersion was extruded by a plastic needle into 2% (*w*/*w*) calcium chloride and was stirred at room temperature. The gel beads were allowed to stand in the solution for 20 min before being separated and washed with distilled water. The beads were dried at 37 °C for 12 h.

#### 3.6.2. Wax-Incorporated Alginate-Based Emulsion Gel Beads

Large amounts of wax (bee wax and carnauba wax) were melted in a water bath at 60–85 °C, depending on the melting range of the waxes used, as adapted from Sriamornsak *et al*. [1,7]. The molten wax was dispersed in a homogenized emulsion mixture of alginate, olive oil and tamarind seed extract at 1% *w*/*w*, which was already heated to the same temperature, and then mixed until a homogenous mixture was obtained. The hot-melted mixture was extruded into 2% calcium chloride (cooled at 5 °C). The wax-incorporated emulsion gel beads obtained were treated in the same manner as the calcium alginate gel beads.

### 3.7. Study of the Particle Size of the Gel Beads

The mean diameter of 50 dried beads was determined by optical microscopy (BH-2, Olympus, Shinjuku-ku, Japan). The microscope eyepiece was fitted with a micrometer, by which the size of the beads could be determined.

### 3.8. Determination of Percentage of Encapsulation Efficiency (% EE)

To determine the % EE of the beads, 500 mg beads was crushed and dispersed in methanol, as described by Gavini *et al*. [11]. The dispersion was sonicated for 15 min, left overnight for 24 h and filtered. A 1 mL sample was taken and diluted with methanol, and the phenolic content assay was performed [12]. The filtrate obtained after bead collection on the filter medium and dilution with phosphate buffer 7.4 was analyzed using a UV-Visible spectrophotometer at 760 nm. Total phenolic content was determined by the Folin-Ciocalteau assay using gallic acid as the standard solution. Sample or standard solution (0.1 mL) was added to a 25 mL test tube containing 8.4 mL of distilled water. Folin-Ciocalteau reagent (0.5 mL) was added to the mixture and shaken for 5 min before adding 1 mL of 20% Na_2_CO_3_ solution. The mixture was shaken before incubating in the dark for 1 h at room temperature. Absorbance was measured against the reagent blank at 760 nm. Total phenolic contents were expressed as milligrams of gallic acid equivalents per g of extract. EE (%) = total phenolic contents in beads/total phenolic content in extracts × 100.

### 3.9. Determination of Active Ingredient Release

The *in vitro* release of tamarind seed extract from the different formulations was examined using a USP dissolution apparatus 1 (Erweka, Heusenstamm, Germany) with 1000 mL of simulated gastric fluid medium (SGF, pH 1.2) and basket rotation at 100 rpm, as adapted from Sriamornsak *et al.* [7]. The temperature was controlled at 37 ± 0.1 °C. Samples were taken at appropriate time intervals and assayed spectrophotometrically (Hitachi U-2000 UV-Visible spectrophotometer, Tokyo, Japan) for total phenolic content. All dissolution runs were performed in triplicate.

### 3.10. Data Analysis

All experimental measurements were performed in triplicate. Results were expressed as the mean value ± standard deviation. Statistical significance in this study was examined using analysis of variance (ANOVA) by the LSD test. The value of *p* < 0.05 was considered statistically significant.

## 4. Conclusions

The water extract of tamarind seed (TSCH) was the most effective due to its greater free radical scavenging activity and toxicity to the mitochondrial membrane of cancer cells. Therefore TSCH was selected for further study of the development of new formulations for the encapsulation and intragastric floating delivery of tamarind seed extract using wax-incorporated emulsion gel beads. The development of tamarind seed extract loaded into emulsion gel beads using a practical approach to control the active ingredient release over an extended period of time was successful. The intragastric floating drug delivery system was prepared by incorporating low density materials, *i.e.*, oils and/or waxes. The incorporation of wax into the beads had an influence on the percentage of encapsulation efficiency and the percentage of active release resulting from the structure of the wax and the diameter of the gel beads. The maximum of 85.43% active ingredient encapsulation efficiency was obtained in the emulsion gel beads containing bee wax, and the percentage of active release from the formulation containing carnauba wax was lower than that from the formulation containing white bee wax. In addition, an increased amount of waxes slowed the release of the active ingredients of tamarind seed extracts from emulsion gel beads. Therefore, this study developed floated beads through the formation of wax-incorporated emulsion gel beads using a modified ionotropic gelation technique to enhance the efficiency and control the delivery of active ingredients that had pH-dependent solubility and instability at intestinal pH. The method could be applied to functional food and nutraceutical products for their antioxidant and anticancer capacity.

## Figures and Tables

**Table 1:** Extraction process, total phenolic content and superoxide anion scavenging activity of tamarind seed coat on various extractions.

Sample	Total Phenolic Content (GAE mg/g Extract)	Inhibition (%)				IC_50_ (µg/mL)
		1000 (µg/mL)	500 (µg/mL)	50 (µg/mL)	5 (µg/mL)	
TSC50	23.69 ± 0.29	90.45 ± 4.87	76.41 ± 0.84	61.08 ± 1.42	44.10 ± 2.76	11.1 ± 0.09
TSC95	43.98 ± 0.33	94.98 ± 0.82	93.58 ± 3.06	75.83 ± 1.47	54.95 ± 0.64	<5
TSCH	39.39 ± 0.19	92.44 ± 1.61	90.67 ± 0.92	77.84 ± 2.22	72.49 ± 0.98	<5

Results were expressed as the averages of triplicates ± S.D.

**Table 2:** Mitochondrial dehydrogenase activity of various tamarind seed coat extracts.

IC_50_ (µg/mL)	Cancer Cells			Normal Cells	
	HepG2	HeLa	Caco2	L929	HK-2
TSC50	>1000	>1000	602.50 ± 130.25	>1000	>1000
TSC95	>1000	>1000	249.21 ± 49.70	>1000	>1000
TSCH	75.27 ± 10.54	15.06 ± 2.54	100.73 ± 11.84	444.16 ± 45.56	>1000

Results were expressed as the average of triplicate measurements ± S.D.

**Table 3:** Effect of the types and concentrations of waxes on the mean diameter and total phenolic content of emulsion gel beads containing tamarind seed extracts.

Gel Beads with Different Types and Concentrations of Waxes	Mean Diameter (mm)	Total Phenolic Contents (GAE mg/g Extract)
Gel bead (No wax)	2.47 ± 0.16	27.01 ± 0.72
Gel bead + Bee wax 1%	2.26 ± 0.08	33.65 ± 1.20
Gel bead + Bee wax 2%	2.23 ± 0.09	35.23 ± 2.24
Gel bead + Bee wax 3%	2.20 ± 0.12	36.26 ± 2.37
Gel bead + Carnauba wax 1%	2.37 ± 0.05	31.56 ± 0.74
Gel bead + Carnauba wax 2%	2.33 ± 0.07	32.57 ± 1.29
Gel bead + Carnauba wax 3%	2.31 ± 0.16	33.61 ± 1.19
